# Functions of disordered eating behaviors: a qualitative analysis of the lived experience and clinician perspectives

**DOI:** 10.1186/s40337-023-00854-4

**Published:** 2023-08-21

**Authors:** Abbigail Kinnear, Jaclyn A. Siegel, Philip C. Masson, Lindsay P. Bodell

**Affiliations:** 1 Department of Psychology, University of Western Ontario, London, ON Canada; 2 Department of Psychology, San Diego State University, San Diego, CA USA

**Keywords:** Eating disorders, Functions, Binge eating, Restricting, Purging, Clinicians, Thematic analysis

## Abstract

**Background:**

One method to improve treatment outcomes for individuals with eating disorders (EDs) may be understanding and targeting individuals’ motives for engaging in DE behaviors—or the functions of DE behaviors. The goal of this study was to investigate and categorize the various functions of DE behaviors from the perspectives of adults who engage in DE behaviors and clinicians who treat EDs.

**Methods:**

Individuals who engage in DE behaviors (*n* = 16) and clinicians who treat EDs (*n* = 14) were interviewed, and a thematic analysis was conducted to determine key functions of DE behaviors.

**Results:**

Four main functions of DE behaviors were identified by the authors: (1) alleviating shape, weight, and eating concerns; (2) regulating emotions; (3) regulating one’s self-concept; and (4) regulating interpersonal relationships/communicating with others.

**Conclusions:**

Differences in participant responses, particularly regarding the relevance of alleviating shape and weight concerns as an DE behavior function, highlight the importance of individualized conceptualizations of DE behavior functions for any given client.

**Supplementary Information:**

The online version contains supplementary material available at 10.1186/s40337-023-00854-4.

## Abstract (plain language summary)

There are many reasons why individuals engage in disordered eating (DE) behaviors, and gathering information on these functions of one’s DE behaviors might help clinicians decide which treatments to administer to individual clients. This study identified and organized numerous functions of DE behaviors based on perspectives of both adults who engage in DE behaviors and clinicians who treat EDs. These functions can be largely grouped into four categories: (1) reducing concerns about one’s shape, weight, and/or eating habits; (2) managing one’s emotions; (3) managing one’s beliefs about themselves as a person; and (4) managing relationships with others or communicating with others. Using treatments that address these reasons for one’s DE behaviors may be beneficial for clients.

**Supplementary Information:**

The online version contains supplementary material available at 10.1186/s40337-023-00854-4.

## Background

Leading treatments for eating disorders (EDs) are ineffective for more than two-thirds of patients with anorexia nervosa (AN) and approximately half of patients with bulimia nervosa and binge eating disorder [1]. Individualized case conceptualizations continue to be a critical part of treatment planning for various forms of psychopathology, including EDs [2, 3]. Considering the specific function that disordered eating (DE) behaviors may serve could potentially improve case formulation and decision-making in ED treatment. Indeed, DE behaviors are often conceptualized as maladaptive behaviors in which an individual engages to attain some goal, suggesting that individuals use DE behaviors because they serve a specific function [4]. Moreover, not all individuals who engage in DE behaviors meet narrow diagnostic criteria for an ED, and regardless of diagnostic status, DE behaviors have an adverse effect on health and well-being [5, 6]. Comprehensively understanding the functions of distinct DE behaviors (i.e., restricting, binge eating, self-inducing vomiting, misusing laxatives, and excessively exercising) may inform strong case conceptualizations and help clinicians determine intervention targets and treatment modalities for those with EDs and those who engage in DE behaviors [4, 7].

In addition to influencing shape and weight, several factors may be important motivators of DE behaviors. DE symptoms may help individuals regulate emotions [8, 9]; escape critical self-awareness [10]; provide a sense of worth and identity [11]; feel powerful and in control [12, 13]; and cope with relational difficulties and communicate with others [14, 15]. For example, a qualitative study aimed at understanding women’s experiences with EDs indicated that DE behaviors can serve as a means of connecting with others and gaining a sense of control [16]. Although this study provides important information on individuals’ experiences with EDs, the study’s focus was not on the nuanced and varied functions that DE behaviors can serve for people, nor did they seek information from clinicians who treat EDs [16]. Given the importance of goal consensus and collaboration in therapeutic relationships [17, 18], it is useful to consider the perspectives of individuals who engage in DE behaviors as well as clinicians who deliver treatment for EDs. Relatedly, Budd [16] exclusively explored social functions of EDs, rather than their potential biological and psychological functions. Given that EDs are biopsychosocial conditions [19], looking beyond just the social functions of DE behaviors is essential for comprehensively understanding these complex conditions.

Multiple qualitative studies have examined both patient experiences of and clinician perspectives on AN. Kyriacou and colleagues [20] conducted focus groups with individuals receiving in-patient treatment for AN, parent caregivers, and clinicians working in the ED unit to explore difficulties with emotions and social cognition in AN. Regarding potential functions of AN, they concluded that individuals may use restriction to avoid and cope with negative emotions [20]. Additionally, Sibeoni and colleagues [21] conducted a meta-synthesis of qualitative studies to examine beliefs about the causes of AN and the experience of AN according to adolescents with the disorder, parents, and healthcare providers. They found that adolescents often described perfectionism, low self-esteem, poor body image, the desire to ‘fit in,’ and identity difficulties as causes of their AN; the adolescents also noted that their AN functioned to protect them, increase their energy and self-esteem, and provide them with feelings of being in control. Healthcare providers cited biomedical causes of AN, and they described a need for control (over others) as the core of the disorder [21]. Although these studies provide insight from various perspectives on the experience and some functions of AN, they did not explicitly seek to understand the functions of the restrictive behavior—rather than the causes and associated difficulties of the full syndrome—and they did not specifically investigate the functions of binge eating and compensatory behaviors.

### The present study

Understanding which functions are the most central for an individual—rather than defaulting to shape and weight concerns as driving individuals’ eating pathology—may be important for treatment selection and decision making. However, to our knowledge, no study has examined the functions of restricting, binge eating, and compensatory behaviors from the perspectives of people who engage in DE behaviors as well as clinicians who treat EDs. We opted to employ a qualitative methodology, because qualitative methods such as semi-structured interviews allow researchers to collect rich data about individuals’ experiences that otherwise might be overlooked with quantitative methods [22]. Using a post-positivism epistemological framework, researchers acknowledge that multiple subjective experiences of reality can exist and that research can never fully describe one truth [23, 24]. The goal of this study was to characterize subjective functions of different DE behaviors from multiple vantage points to help inform more comprehensive, personalized case conceptualizations in ED treatment. Given that data source triangulation is a well-established mode of enhancing the credibility of qualitative research (see [25, 26]), we aimed to achieve this by conducting semi-structured interviews with both clinicians who treat EDs (i.e., subject matter experts) and individuals who engage in DE behaviors (i.e., experts of their own experiences).

## Methods

### Participants

**Fig. 1:**
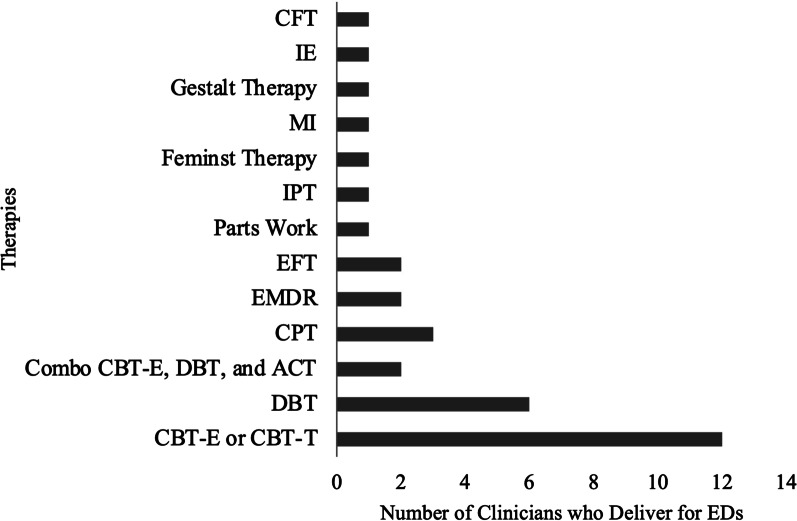
Therapies administered by clinician participants*.* These therapies are not mutually exclusive; CBT-T = CBT-Ten; DBT = Dialectical Behaviour Therapy; ACT = Acceptance and Commitment Therapy; CPT = Cognitive Processing Therapy; EMDR = Eye Movement Desensitization and Reprocessing; EFT = Emotion Focused Therapy; IPT = Interpersonal Psychotherapy; FT = Feminist Therapy; MI = Motivational Interviewing; IE = Intuitive Eating; CFT = Compassion-Focused Therapy

Participants were individuals who self-identified as engaging in DE behaviors (*n* = 16) and clinicians who treat EDs (*n* = 14). Participants were recruited using various remote methods, such as advertisements on [Masked] University Facebook groups and a Reddit page dedicated to EDs. Advertisements were labeled either, “Do you often go to extreme measures to control your shape and weight?” or, “Have you or someone else thought you have an eating disorder?” and indicated that we were seeking “individuals who engage in eating disorder behaviors (such as restricting food intake, binge eating, and purging).” A member of the research team emailed clinicians across Canada, and snowball sampling methods were used to recruit additional clinician participants. Table 1 includes participant demographic information, and Fig. 1 includes clinician theoretical orientation.

**Table 1:** Descriptive statistics among total sample

	N	M/%	SD	Range
ED group	16			
Age	16	24.38	5.19	18–32
Gender				
Woman	14	87.5		
Man	1	6.25		
‘Uncomfortably female’	1	6.25		
Race/ethnicity				
White	12	75.00		
Asian	1	6.25		
Black or African American	1	6.25		
Middle Eastern	1	6.25		
Multi-racial	1	6.25		
Body mass index (BMI)	16	23.00	8.10	17.9–51.7
Behaviors endorsed				
Restriction	16	100.00		
Binge eating	14	87.50		
Compensatory behaviors	10	62.50		
Clinician group	14			
Profession				
Clinical or counselling psychologist	4	28.57		
Social Worker	4	28.57		
Dietician	2	14.28		
Psychiatrist	2	14.28		
Registered psychotherapist	2	14.28		
Years of experience	14	12.60	11.90	1–39

### Procedures and materials

This study received approval from [Masked] University’s Research Ethics Board, and all participants provided informed consent. Participants completed a semi-structured interview as part of a larger study. These interviews were conducted via telephone due to COVID-19 pandemic restrictions on in-person research, which allowed us to recruit participants internationally. Interviews were audio-recorded, and extensive notes were taken during each interview. All participants were given the option to enter their contact information in a raffle to win one of six $25 prizes.

We first asked participants who engage in DE behaviors if they restrict their food intake, engage in binge eating, self-induce vomiting, use laxatives, and/or engage in excessive exercise. We asked participants to describe what each behavior “looks like” for them to understand the presentation of their behaviors. Although we did not conduct formal diagnostic interviews, nearly all participants described pathological eating behaviors. Subsequently, we asked participants “Can you tell me all the reasons you might engage in each of these behaviors?” and “Why do you think other people might do this that may not apply to you?” We asked clinician participants, “What do you think are the reasons people with eating disorders engage in the following behaviors: restriction, binge eating, self-induced vomiting, laxative use, and excessive exercise?” Interview guides are provided (see Additional file 1).

Sample size was guided by the principle of information power, such that fewer participants are needed when the data contain more relevant and rich information data [27]. Data covered a breadth of DE functions, with many participants describing functions in great detail. Additionally, throughout the interviews, many responses were repeated [28]. Interviews averaged approximately 23 min for the DE behavior group and 20 min for clinicians. Two research assistants (RAs) transcribed each semi-structured interview verbatim using Microsoft Word. A third RA combined and reviewed the transcripts. When RAs found discrepancies between the transcripts, the RA combining the transcripts consulted the audio-recorded interview.

#### Researcher positionality

Researcher positionality plays a critical role in shaping the way data are generated, analyzed, and presented [29, 30]. Therefore, it is essential that we position ourselves in relation to this analysis. The first author was responsible for study design, conducting interviews, data analysis, and manuscript writing. The second and third authors served as consultants to the project and assisted with manuscript editing. The last (“senior”) author oversaw the project and participated in study design and manuscript writing. All authors identify as White, cisgender, and able-bodied. The third author identifies as a man and the other authors identify as women. At the time of the study, the first two authors were graduate students, the third author was a clinician, and the fourth author was an assistant professor, all in Southwestern Ontario. Together, the authors have expertise in qualitative and quantitative methods as well as EDs and treatment through lived experiences and/or education and training. Having these experiences as people with lived experiences of EDs and/or clinicians provides us with a unique, though incomplete, lens through which we view these data. While our varied experiences tune us in to different features of the interviews, our relative homogeneity may have resulted in gaps in our analysis.

### Data analyses

The first author reviewed all 30 sets of interview notes and created a list of 66 DE behavior functions using participant responses. Some of these functions were similar in nature, but they were all included to capture the different language participants used. This method yielded a total of 158 potential functions of DE behaviors when combined with functions from our research team and extant literature on functions of maladaptive behaviors (e.g., [4, 31–33]) The first author sorted all functions into overarching categories and subcategories using her knowledge of theories and mechanisms known to be related to EDs. The first author, last author (i.e., principal investigator), and two other lab members discussed and agreed upon these categories. We resolved disagreements in categories by referring to the principal investigator’s knowledge and expertise in the field of EDs and by coming to verbal agreement. Although agreement on these categories does not fully represent objective reality (i.e., [23, 24]), we sought verbal agreement to achieve researcher triangulation. This framework became the codebook for the thematic analysis of the semi-structured interviews.

We conducted a codebook thematic analysis on the semi-structured interviews, using both a semantic and latent analytic approach (i.e., examining both overt and underlying meanings of participant responses) and a critical realist framework [34–37]. Using the codebook, two RAs coded each interview. RAs took notes as they were coding and indicated any responses that did not fit within the identified themes. A third RA reviewed initial codes in the transcripts to identify any additional codes that were not noted by the other RAs. After coding was completed, a fourth RA collated all coded sections from the Word documents into a Microsoft Excel sheet. The first author and coding RAs held consensus meetings to ensure sufficient agreement on the categorization of participants’ responses and to resolve any inconsistencies. Once the codes were determined, the research team identified the themes of the data.

The thematic analysis adhered to the trustworthiness criteria of credibility, transferability, dependability, and confirmability outlined by Lincoln and Guba [38], and we established trustworthiness at multiple stages of data analysis, following recommendations from Nowell and colleagues [26]. For instance, researcher triangulation occurred at many stages of the analysis, including codebook development, discrepancy identification, and code determination, and the process of coding and analyzing data has been described above in detail. Finally, because analyses were conducted on the spectrum between latent and semantic analyses [34], RAs often coded responses based on participants’ explicit statements. Through a post-positivist lens, we acknowledge that our individual perspectives shaped the analysis of the data, and therefore, the following analysis is a co-construction of participants’ motivations of eating disorder behaviors.

## Results

**Fig. 2:**
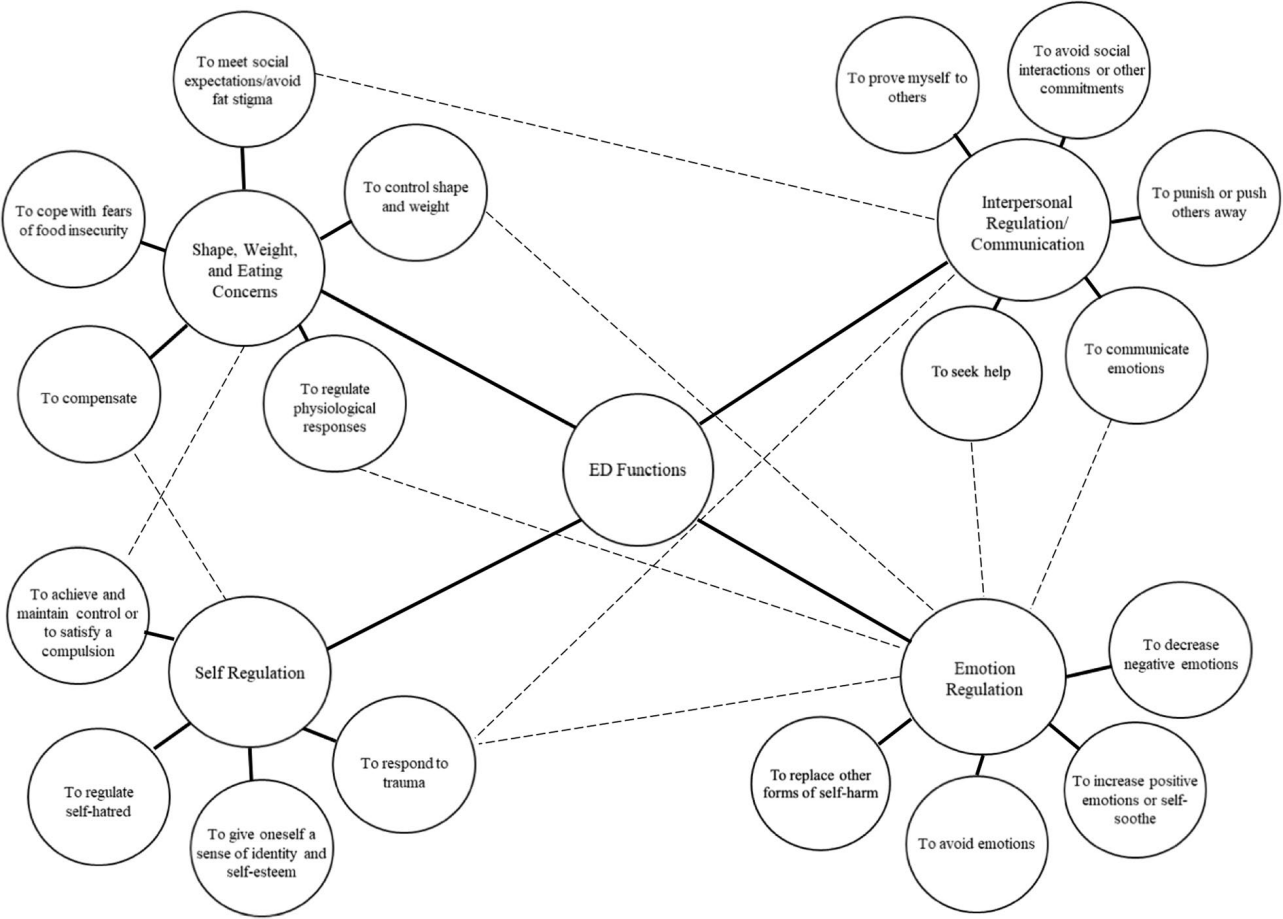
Themes constructed from interviews with 16 participants who engage in DE behaviors and 14 clinicians. Dashed lines represent functions that overlap between categories

Through the analytic process, we identified four key themes that characterize the functions of DE behaviors from the interviews: (1) alleviating shape, weight, and eating concerns; (2) regulating emotions; (3) regulating one’s self-concept; and (4) regulating interpersonal relationships/communicating with others. Each of these four themes was divided into subthemes of more specific functions (Fig. 2).

Although each of these themes was well-represented in the data, some themes were more frequently mentioned than others. Frequency counts and examples of each subtheme are included in Table 2. Although frequency counts do not capture how prominent themes were in each interview [39], we have included them to demonstrate differences we coded in responses between clinicians and people who engage in DE behaviors.

**Table 2:** ED function themes, frequency counts, and example quotes

Theme	Subtheme	ED Group (n)	Clinician (n)	Example
Alleviating shape, weight, and eating concerns	To control shape and weight	13	14	*Not so much now, but when I was younger, I definitely used my eating disorder to control my weight (ED Group Participant #9)*
				*Surface level ones would be managing weight, and I think that that’s, if you would ask anybody who’s kind of dealing with ED stuff and views of body image that would probably be on the top of their mind like, ‘I restrict because I want to be thin or I purge because I want to be thin’ and so that’s certainly like a very, like surface level reason people do it but I think that there's a lot more under that (Clinician #9)*
	To meet societal expectations/avoid fat stigma	13	11	*It feels like I have to do it…I do have this huge fear of being overweight or obese, like I just, I do. I don't want to ever admit that, but I have that fear (ED Group Participant #15)*
				*It’s just external versus internal, so there’s internal reasons that someone would want to lose weight, but then also people can have external. So by like parents, family members, partners telling them they should lose weight or saying disparaging comments about their weight and so by doing things like restricting or vomiting or exercise or laxatives they feel that like relief by kind of doing what other people and kind of want or expect or, or what they think they should do (Clinician #1)*
	To regulate physiological responses	8	7	*But the bingeing that came afterwards, like it really was just a matter of like a consequence of the restricting…it’s like, your body is saying like ‘we need this food, like we, it’s been way too long’ (ED Group Participant #13)*
				*It may look like the binge eating is driven by stress or you know, ‘I'm an emotional eater or I had trauma when I was three.’ I don't actually agree with those. In most cases, I don’t agree that that's the cause. I think …it’s starvation, that then makes you susceptible to…not requiring many triggers…to binge eat (Clinician #6)*
	To compensate for other behaviors or lack thereof	6	10	*So, instead of restricting for so long it was like, ‘Great I can eat this at dinner time and just throw it back up’ or ‘I can eat the food that I was restricting for so long that I didn’t allow myself to have and then just purge it right back up’ (ED Group Participant #13)*
				*They perceived to have eaten too much, and lost control of their eating, so they’re going to make up for it by getting rid of the food to compensate for that (Clinician #3)*
	To cope with fears of food insecurity	0	3	*Growing up, if food was restricted or…if you were food insecure and food was not readily available, those are big precursors to eating disorders, because just the thought of not having food and then having it in abundance can be really hard to handle. Or if you were always told like you can't eat or when to eat or what to eat, it's really hard to manage that once you’re on your own and have food available (Clinician #11)*
Emotion regulation	To decrease negative emotions	16	13	*Typically, I binge and purge for stress related reasons. So, when I was younger when I first started being bulimic like when I was in middle school it was primarily because of stress at home, and then when I got a little older it was because of school stress, and then as I got even older now it's work stress that kind of feeds my—it's kind of like the relief of it…it's a bad coping skill, but it's like, it's the most effective coping skill for me, even though I’ve like tried to break this habit and gone to treatment (ED Group Participant #9)*
				*I think that people are more likely to talk about [anger] at themselves, but sometimes, sometimes they'll say it's just about others, like you know a family member, or person that has a relationship with… or it could be something, you know it could just be their situation (Clinician #8)*
	To increase positive emotions or self-soothe	9	12	*I felt incredible when I was restricting and when I was bingeing or had to use laxatives, I felt weaker (ED Group Participant #7)*
				*They can count on [the eating disorder], and it's hard for people to relinquish that, and to lose it can feel like a real significant loss to people when they decide to work on their eating disorder (Clinician #7)*
	To avoid emotions	7	12	*I need to slow my brain down, like nothing else is working to do this. So, I can completely numb out by like bingeing and purging my face off (ED Group Participant #8)*
				*As long as someone is thinking about calories or ‘Should I have eaten this?’ or ‘Should I not have eaten this?’ or ‘Should I have thrown up?’ or ‘Will I throw up?’ all those things, they aren't thinking about other issues (Clinician #10)*
	To replace other forms of self-harm	2	0	*Originally, when I first started, it wasn't the case. But now, it’s the case that it also helps prevent me from relapsing into drug use…And it's come back periodically but this latest one is absolutely driven partly, like, as a way to cope with life without going into drugs (ED Group Participant #12)*
Self-regulation/maladaptive schema response	To achieve and maintain control or to satisfy a compulsion	16	13	*It just feels like I couldn't, I tried to imagine not, and I just can't (ED Group Participant #15)*
				*I have a number of clients right now who feel almost restriction in other areas, like they have very domineering parents, for example, very domineering partner, but [the ED behavior] is something that they believe is within their control, even though psychologically it often is outside of their control, once they begin (Clinician #2)*
	To respond to trauma	6	12	*Part of it could be just, feeling small, wanting to be small… it just matches the mental like, playground of the world that I have…like I, if you don’t feel significant, then and part of like I guess an almost OCD reaction is like if I'm not going to feel significant, I'm gonna go all the way (ED Group Participant #12)*
				*Trauma is a huge part of it too right, like that feeling of lacking safety or autonomy or control of your own body can certainly lead to ED behaviors, because you know restricting, purging, bingeing, all these different things can totally numb us so it’s a protective way of managing our trauma (Clinician #9)*
	To give oneself a sense of identity and self-worth	5	8	*Staying small or not eating it—turned very competitive. Where if it was me hanging out with some friends at school and one of them was complaining about being so hungry because they haven't eaten all day, in my head, I would either feel bad because I did eat that day, or I'd feel proud because I didn't eat at all that day and I feel fine…I would feel better than them to an extent because I had the willpower not to eat (ED Group Participant #7)*
				*I'm not good at anything else, but I can sure count calories well (Clinician #12)*
	To regulate self-hatred	7	5	*I’m just generally always feeling like an extremely ugly person and thinking that I like deserve to not eat because I'm that ugly kind of thing (ED Group Participant #10)*
				*I think that [the eating disorder] sort of plays out different maladaptive schemas. So, if I feel like I am defective… it could be that I…punish myself with behaviors or that it somehow compensates for those maladaptive schemas. Then I also think that those, those cognitions sort of…in a cognitive behavioral way, perpetuate… ‘I'm not good enough,’ that may perpetuate the cycle of, ‘so therefore I shouldn’t eat this meal, because I, my body doesn't look good,’ I don't know and, ‘and then I feel better about myself’ (Clinician #12)*
Interpersonal regulation and communication	To seek help	7	8	*The um, external appearance of like, ‘I'm not okay,’… can be a way to try and breakdown that barrier of like, ‘I don't know how to ask for what I need, but my body will ask for it without me having to use my words’" (ED Group Participant #8)*
				*Restriction…I think it can also be a way of trying to subconsciously influence relationships so…wanting to maybe, again subconsciously play the sick role in order to have people maybe engage with them more or control kind of a relationship by kind of subconsciously engaging in symptoms in order to manage that relationship (Clinician #5)*
	To avoid social interactions or other commitments	5	5	*Sometimes I use it as an excuse of like well like I'm crazy and like super eating disordered, so there's no reason for me to bring another person into a relationship (ED Group Participant #8)*
				*Especially in university, people use the eating disorder behaviors to procrastinate (Clinician #10)*
	To communicate emotions	4	5	*If it’s like, ‘Well I want to eat here,’ and they’re like, ‘No’, then I can just be like, ‘Well fine, since you make me feel fat for wanting to eat there, I just won't eat at all’ (ED Group Participant #7)*
				*I think it's a way to communicate anger and sort of an ‘eff you’ to people, and so if I sort of binge and purge in my spouse's bathroom or something like that, you know, it's a way to kind of communicate that message and push them away in that way. Or I guess it could activate them…I think that…it is a form of communication, and with that form of communication it can either pull people in or push them away (Clinician #12)*
	To prove oneself to others	2	3	*I felt like in that relationship I consistently had to like prove that like I had some sort of like self-control and like maturity because I was dating somebody older than me and like I felt like he…was like testing my ability to like be ready to date a man his age. And he never like specifically said those things…but he was like very obvious and like the way that things went down (ED Group Participant #11)*
				*I don’t need anything, I am self-sufficient, not only do I not need people and help but I don’t even need food, and I don’t even want it like you guys do" (Clinician #13)*
	To punish or push others away	1	3	*They would make me feel worthless, so I want to punish them by punishing myself, because I knew they would feel guilty over me punishing myself (ED Group Participant #7)*
				*I think I often see, thinking about attachment, there can often be like a kind of a push–pull dynamic of interpersonal communication with individuals. We actually see a lot of this play out in the treatment world …There’s kind of like a push, you know, ‘I want to be loved so much so like eating disorder brings people in but then as like when people get too close there’s like a push away of like please don’t come near me,’ and the eating disorder kind of ends up being like an ‘eff you’ or a way to create distance relationally. So there’s kind of a push–pull pattern interpersonally with the client with an eating disorder and the function of like negotiating safety in relationships (Clinician #14)*

### Shape, weight, and eating concerns

The first key theme surrounding the functions of DE behaviors is the role of shape, weight, and eating concerns. Functions within this theme directly relate to the appearance-related goals and physical consequences of DE behaviors.

#### To control shape and weight

We identified the desire and need to control one’s shape and weight as one of the most frequently reported functions of DE behaviors by participants in the DE behavior group and clinicians. Moreover, three participants in the DE behavior group and one clinician mentioned that changing shape and weight is the original reason for engaging in DE behaviors, suggesting this function may become less relevant as the behaviors progress. While some clinicians firmly believed that this is the key underlying function or core of DE behaviors, others did not agree. Rather than being the true core of EDs, changing shape and weight may also be the “surface-level” reason for engaging in DE behaviors (Clinician #9).

#### To meet societal expectations and avoid fat stigma

The goal of meeting societal standards (for women) and avoiding fat stigma was closely linked to the function of changing shape and weight, although this function was mentioned in the context of external cues and expectations. Clinicians and participants in the DE behavior group mentioned that engagement in DE behaviors helps individuals keep up with societal standards and female body ideals, some of which was reinforced through positive feedback from others in their lives. Some participants in the DE behavior group specifically noted their desire to avoid becoming fat or “obese;” they reported that they did not want to be the largest person in their friend group or “*turn out like [their family members who are ‘overweight’]”* (DE behavior group Participant #11).

#### To regulate physiological responses

Individuals may also engage in DE behaviors to regulate uncomfortable physiological responses, which are often a result of DE behaviors. Participants in the DE behavior group mainly discussed the regulation of physiological responses in the context of binge eating due to hunger. Clinicians echoed this sentiment, and they also described purging behavior (i.e., vomiting and using laxatives) to relieve gastrointestinal distress or the distress that may follow eating as well as to feel physically empty and to decrease bloating.

#### To compensate for other behaviors or lack thereof

Some individuals with DE behaviors noted that their behaviors functioned to compensate for binge eating or to punish themselves for not restricting or exercising enough. When people who engage in these behaviors “fail” to meet their behavioral goals, they might compensate by restricting more or punish themselves through binge eating. Additionally, some participants—DE behavior group and clinicians—framed self-induced vomiting as a way to live a “normal” life, free from intense restriction and the symptoms that accompany restriction (i.e., dizziness, weakness).

#### To cope with fears of food insecurity

A few clinicians (but no participants with DE behaviors) noted that individuals may engage in DE behaviors because they fear future inaccessibility of food. Clinicians speculated that beliefs about food scarcity could have been the result of poverty and/or views of food in the house growing up.

### Emotion regulation

The next prominent theme we identified in the data is emotion regulation. All 30 participants mentioned emotion regulation as a function of DE behaviors in the interviews.

#### To decrease negative emotions

Many participants described DE behaviors as decreasing stress and/or anxiety, with participants in both groups noting the use of DE behaviors to decrease specific negative emotions, such as loneliness, anger, guilt, sadness, disappointment, and boredom.

#### To increase positive emotions or self-soothe

Multiple clinicians and participants in the DE behavior group mentioned that a function of DE behaviors is to comfort oneself. Clinicians added that DE behaviors are something that people can always count on to make them feel better when times are tough. However, the ways in which each behavior contributes to positive emotions may differ. For example, some participants reported that binge eating feels comforting, like “a warm hug” (Clinician #12), whereas restricting might help to “mellow out” (DE behavior group Participant #16), provide a feeling of safety, or increase their energy and mood. Clinicians noted that eating, exercising, and vomiting can be positively reinforced through reward (i.e., endorphin release) and feelings of stimulation.

#### To avoid emotions

Another function that DE behaviors serve is to avoid emotions completely. This is often accomplished through using the DE behaviors as a distraction and to make oneself feel numb. Some participants noted that restriction and exercise could be solutions to stress by making one emotionless. Clinicians often said that DE behaviors serve to numb and trigger a state of dissociation, with some drawing comparisons to self-harm.

#### To replace other forms of self-harm

A less common function of DE behaviors was to replace other harmful behaviors and/or serve to tolerate distress. Two participants in the DE behavior group highlighted that their DE behaviors prevent them from using substances, seemingly as a form of harm-reduction.

### Self-Concept regulation

Another overarching theme of DE behavior functions we identified is self-concept regulation or responding to maladaptive schemas. Individuals who engage in DE behaviors may have strong negative beliefs about themselves and their place in the world. Engaging in DE behaviors can help them justify, cope with, and make sense of those beliefs.

#### To achieve and maintain control or to satisfy a compulsion

Both clinicians and participants in the DE behavior group frequently said that DE behaviors function to establish control over something in one’s life when they do not have control over other aspects of their lives, often using this exact wording.

Another commonly cited function of DE behaviors within this subcategory is to satisfy a compulsion or continue a habit. For instance, one participant in the DE behavior group stated:

*If someone has an eating disorder, like you kind of get into those compulsive behaviours and it just, you get set in your ways and that's what makes it so difficult to break out of. So, I feel like sometimes if someone were to get like stuck in those compulsive ways, like ‘oh I'm gonna eat this only’ or ‘I'm gonna* *do this,’ like all these eating disorder rules, then I think that like once you get trapped in that cycle then it, it just kind of happens* (ED Group Participant #9).

One clinician commented on this function by noting that DE behaviors do “work” for people in terms of meeting certain needs; when they work, they become more entrenched and embedded over time (Clinician #8). When this occurs, the DE behaviors become their default coping strategy. However, one clinician specifically pushed against this idea, claiming that people often say EDs are used to maintain control, but that this can, at times, mask an underlying need to specifically control shape and weight.

#### To respond to trauma

Many participants stated that DE behaviors might be a response to trauma or method for managing trauma. Many people with DE behaviors gave this response for reasons why others might engage in DE behaviors. For instance, some might engage in DE behaviors as an attempt to separate themselves from their body that was abused. Clinicians often mentioned EDs as a means of creating safety either physically (i.e., through making one’s body larger or smaller) or mentally (i.e., through numbing oneself). One clinician phrased it as, “*We don’t feel we’re going to be hurt in the dissociative state*” (Clinician #2).

#### To give oneself a sense of identity and self-worth

Both participants who engage in DE behaviors and clinicians stated that DE behaviors can give someone a sense of identity and self-worth. Participants in the DE behavior group described how DE behaviors are something they can be the best at, and the behaviors can provide a sense of “*purpose or an identity when you don’t have a whole lot going on in your life or feel like overwhelmed*” (DE behavior group Participant #16). That is, individuals may find a sense of identity through engagement in DE behaviors. However, identification with controlling shape, weight, and eating can also dominate and likely shrink other parts of identity [40].

#### To regulate self-hatred

People who engage in DE behaviors and clinicians both discussed DE behaviors as a method of relieving thoughts of self-hatred or preoccupying individuals until the thoughts dissipate. Some clinicians mentioned that DE behaviors can serve self-harm purposes. Notably, however, some clinicians did not agree that this is a function of DE behaviors, with one stating that there are many ways that people damage and punish themselves that are purely aversive, whereas food has some rewarding aspect to it.

### Interpersonal regulation and communication

The final theme of DE behavior functions we identified was the goal of interpersonal regulation/communication. DE behaviors can be a way for individuals to manage their relationships and communicate with others. Importantly, clinicians were cautious to describe this as “non-manipulative” or non-malicious; participants in the DE behavior group did not explicitly state this.

#### To seek help

Both participants in the DE behavior group and clinicians discussed how DE behaviors can be a method of asking for help or communicating that an individual is “not okay.” Clinicians described how DE behaviors (and potential weight loss that might accompany the behaviors) can create a sense of worry and concern in others. Indeed, some clinicians specifically described the DE behaviors as a means of seeking care and support and to have their needs met.

#### To avoid social interactions or other commitments

Both groups noted that DE behaviors can become a way to avoid sexual objectification or attention. Relatedly, DE behaviors were reported to prevent individuals from vulnerability, intimacy, and relationships. Participants also discussed how DE behaviors are used to procrastinate or avoid responsibilities/commitments. Specifically, becoming the “sick person” lowers everyone’s expectations for those with EDs (DE behavior group Participant #16). Together, DE behaviors may enable those who engage in them to “get out” of uncomfortable situations (DE behavior group Participant #13).

#### To communicate emotions

Clinicians and participants in the DE behavior group added that EDs serve to communicate one’s emotions. One participant in the DE behavior group said that they use their DE behaviors to communicate their anger. Clinicians noted that the DE behaviors can be a voice for clients when they feel like they cannot otherwise communicate pain, distress, and difficult emotions.

#### To prove oneself to others

Participants in the DE behavior group said restriction can be used to prove to others that they have self-control. Participants in this group also endorsed that one function of DE behaviors is to prove to others they do not need to rely on anyone for help.

#### To punish or push others away

A few people who engage in DE behaviors noted that the DE behaviors can feel like a form of revenge towards their parents. On the other hand, clinicians spoke more about pushing others away than punishing them.

## Discussion

The goal of this study was to gain a more comprehensive understanding of the functions of DE behaviors from perspectives of individuals who engage in DE behaviors and clinicians who treat EDs. We identified four main functions of DE behaviors: (1) alleviating shape, weight, and eating concerns; (2) regulating emotions; (3) regulating one’s self-concept; and (4) regulating interpersonal relationships or communicating with others. Although most subthemes were cited by both groups, one subtheme was only mentioned by clinicians (i.e., to cope with fears of food insecurity), and one was only mentioned by participants in the DE behavior group (i.e., to replace other forms of self-harm). Additionally, we found that some subthemes overlapped with other themes. For instance, participant responses regarding control were often related to control over shape and weight, and many negative emotions participants described are implicitly related to social contexts (e.g., loneliness, anger, guilt, disappointment, etc.). We also identified tension in both participant groups regarding the primacy of shape and weight concerns as functions of DE behaviors, such that some participants in each of these groups claimed these functions are the core functions of DE behaviors while others in each group noted the opposite.

The tension surrounding shape and weight concerns as the primary function of DE behaviors may exist for several reasons. First, the general public has a skewed view towards media and the perpetuation of unrealistic body standards as a cause of EDs [41]. Some people with DE behaviors may be hesitant to report that regulating shape/weight is a function of their DE behaviors as they may not want to appear hyper-concerned about their appearance (e.g., [42]). Second, clinicians’ theoretical orientations may relate to their endorsement of shape and weight concerns as the primary function of DE behaviors, with cognitive behavior therapy-enhanced (CBT-E) practitioners emphasizing the importance of shape and weight concerns given CBT-E’s focus on overevaluation of shape, weight, and their control as a core feature of EDs [43]. However, clinicians in the study were not explicitly asked to rank the prominence of DE behavior functions, so many did not specify whether they believed shape and weight concerns were the key functions of EDs. Third, it is likely that functions vary both between and within individuals. For example, the function of one’s DE behaviors may be to regulate emotion, and the function of another’s DE behaviors may be to change their shape and/or weight. Finally, it is also possible that the initial function of DE behaviors is to change shape and weight, and that the functions change over time. This explanation is consistent with habit formation theory [44] where we may expect that individuals in earlier stages of their ED may be more likely to engage in DE behaviors to alleviate shape/weight concerns, whereas someone later in their illness may be more likely to engage in DE behaviors to satisfy a compulsion.

Another aim of this study was to learn more about ED-specific functions. For instance, this study highlights the variety of reasons/motivations underlying changing shape and weight, from alleviating concerns regarding societal and personal beauty standards to creating personal safety or avoiding attention from others. Furthermore, the finding that individuals may engage in DE behaviors to cleanse or purify oneself, “to disappear,” and to avoid intimacy provides further support for the idea that DE behaviors may arise to help an individual cope with trauma [33, 45]. Strong correlations between trauma history and EDs have been well-documented, with posttraumatic stress symptoms [46, 47] and accompanying maladaptive beliefs about oneself being potential maintenance factors of DE behaviors [48]. In particular, body shame, low self-esteem, and avoidance of intimacy or future violence may mediate the relation between sexual trauma and DE behaviors [49, 50], and the behaviors may also serve to reduce tension associated with the trauma [50]. Additionally, the function of control seems to be quite prominent for DE behaviors [16, 51, 52]. Finally, some individuals’ DE behaviors may be directly related to gastrointestinal symptoms, and DE behaviors may be perpetuated because they relieve gastrointestinal distress (e.g., [53]). Although we identified some unique functions of DE behaviors, this study adds support to known functions of maladaptive behaviors, such as emotion regulation and interpersonal regulation/ communication [54, 55].

### Clinical implications

Taken together, these data highlight the diversity of functions that DE behaviors serve, with individual differences across participants. The four major themes provide support for the maintenance factors in the transdiagnostic model of CBT-E “broad” (i.e., core low self-esteem, perfectionism, mood dysregulation, and interpersonal difficulties; [15]), and the various subthemes add deeper conceptual understandings. Additionally, case conceptualization is a critical piece of treatment. One aspect of case conceptualization for DE behaviors includes identifying clients’ motives to engage in them, or what need the DE behaviors fulfil for each individual; identifying the specific reasons an individual may engage in specific DE behaviors and matching other treatments to these functions could potentially improve outcomes. For instance, Interpersonal Psychotherapy focuses on interpersonal difficulties rather than on ED symptoms and is the leading alternative treatment to CBT-E for non-underweight patients [43, 56]. For clients whose DE behaviors serve interpersonal functions, Interpersonal Psychotherapy may be more effective at addressing the causal mechanism underlying their behaviors. Conversely, many people with EDs experience elevated shame and self-criticism, which may best be treated by Compassion-Focused Therapy for EDs or mindfulness-based therapies [11, 57]. Clients whose DE behaviors function to regulate trauma responses may benefit from treatment such as Cognitive Processing Therapy [58], Prolonged Exposure [59], Michael White’s Narrative Therapy [60], or integrated cognitive-behavioral therapy for co-occurring ED and posttraumatic stress disorder [61]. Furthermore, even when shape and weight concerns are the primary motivator of one’s DE behaviors, clients and clinicians learning more about fat acceptance/activism may help address fat phobia, and feminist perspectives may be beneficial to people who believe they must conform to societal standards. Similarly, teaching clients they can tolerate the discomfort of living in a larger body or engaging in deliberate fat embodiment might address fear of weight gain [62, 63].

### Strengths and limitations

Limitations of this study include the relatively few participants in the DE behavior group who engaged in compensatory behaviors and the demographic homogeneity of the sample. Indeed, participants in the DE behavior group were primarily adult white women; thus, functions that may be more relevant to individuals from minoritized and marginalized groups, men or individuals of other genders, and children/adolescents may not have been identified in this study, limiting the breadth and generalizability of findings. Therefore, this study is a first step toward comprehensively understanding the functions of DE behaviors. It is also important to note that we interviewed people with DE behaviors and clinicians who treat EDs, so participant groups may have been reporting about different behaviors and levels of severity. With that said, not all individuals who engage in DE behaviors meet narrow diagnostic criteria for an ED, and participants in the DE behavior group may never have had the opportunity and resources to receive a diagnosis. Additionally, we did not collect data on whether clinicians had lived experience with an ED, so it is possible that some clinicians may have had lived experience and responded through that lens. We also did not collect demographic data on the clinicians. Finally, although a unique feature of this study is the direct investigation of DE behavior functions, it is possible that this concept did not resonate with participants. To generate a richer dataset, it would have been beneficial to explore with participants the assumption that DE behaviors serve some function for them. Similarly, given the targeted focus on DE behavior functions, we did not collect additional data on participants’ other lived experiences, such as substance abuse or trauma history, which may uniquely affect the functions of DE behaviors.

Despite these limitations, this study has several strengths, including the inclusion of people who engage in DE behaviors and clinicians who treat EDs from across Canada and internationally. Taking both these “insider” and “outsider” perspectives provides a richness in the data that might have been overlooked otherwise [64]. Clinicians had a range of experiences in terms of their professions, treatment approaches, and their years of experience. Future research should focus on individuals from underrepresented groups to gain an even greater understanding of the functions of DE behaviors and implications for treatment. Additionally, future research should systematically measure these functions of DE behaviors to understand whether they provide unique ways of understanding the development, maintenance, or treatment of EDs.

## Conclusion

This study demonstrated numerous reported functions of restricting, binge eating, and compensatory behaviors, according to individuals who engage in DE behaviors and clinicians who treat EDs. We condensed and organized these functions into four themes and corresponding subthemes. All participants acknowledged that DE behaviors serve *some* function with the specific functions dependent on the individual. Even if these unique functions are not essential mechanisms of the behaviors, identifying functions within these themes and linking them to treatment in a meaningful way may be validating for people with EDs and enhance treatment engagement. Additionally, given that many individuals with EDs do not maximally benefit from treatment, addressing other needs or functions served by DE behaviors may improve treatment outcomes.

### Supplementary Information

**Additional file 1**: Interview guides used for participant interviews.

## Data Availability

The datasets generated and/or analysed during the current study are not publicly available as this could compromise participant privacy, but anonymous data are available from the corresponding author on reasonable request.

## Abbreviations

CBT-E: Cognitive behavior therapy-enhanced
ED: Eating disorder
RA: Research assistant
AN: Anorexia nervosa
