# Supplementary material for: Functions of disordered eating behaviors: a qualitative analysis of the lived experience and clinician perspectives
Source: J Eat Disord. 2023 Aug 21;11:141. doi: 10.1186/s40337-023-00854-4 (PMC10440936; doi:10.1186/s40337-023-00854-4)
Supplement: Supplementary file 1 — Additional file 1: Interview guides used for participant interviews. [file 40337_2023_854_MOESM1_ESM.docx]

**Additional File 1**

ED Behavior Group Interview Script:

We are interested in learning more about the reasons why people engage in disordered eating behaviours, or ways of eating that cause problems for you. Some people intentionally restrict their food intake or eat very little food on purpose. Some binge eat, or experience a loss of control and eat a lot of food in a short period of time - much more than their bodies need. Some people purge or get rid of their food by throwing it up, taking laxatives, or exercising too much. In order to assess the reasons why people do these behaviors, we want to understand what restricting, binging, and purging *do* for you - what functions do these actions serve. We are developing a measure that will ask individuals to check off and rate all the functions that are relevant to them. Before we make the list of these functions, we want to ask you about your perspective on why you engage in specific behaviours. First, I would like to understand which behaviours you engage in.

**Do you restrict your food intake?**

Participant responds Yes or No:

If yes: **Can you tell me what this looks like for you?**

**Do you engage in binge eating, in which you eat a large amount of food in a short period of time and feel a sense of loss of control while eating?**

Participant responds Yes or No:

If yes: **Can you tell me what this looks like for you?**

**Do you self-induce vomiting?**

Participant responds Yes or No:

If yes: **Can you tell me what this looks like for you?**

**Do you use laxatives?**

Participant responds Yes or No:

If yes: **Can you tell me what this looks like for you?**

**Do you engage in excessive amounts of exercise?**

Participant responds Yes or No:

If yes: **Can you tell me what this looks like for you?**

**Regarding each of these behaviours that you said “yes” to, can you tell me all the reasons you might engage in each of these behaviours? You can list reasons that are relevant to one or multiple of the behaviours.**Participant responds:

**Prompt (if necessary):**

**“**Examples of these reasons might be to lose weight, to regulate your emotions, or to punish yourself.”

**“Are there any other reasons you can think of which might lead you to engage in any of the eating disorder behaviours you discussed?”**

**Why do you think other people might do this that may not apply to you?**Participant responds:

**Finally, do you think that the functions of eating disorder behaviours have changed for you (and/or others) during COVID-19? That is, do you think you (and/or others) might engage in these behaviours for different reasons during this time?**

Participant responds Yes or No:

If Yes: **How do you think these functions have changed?**

Participant responds:

**Have you received treatment for your eating disorder? Yes or No**

**If yes: Please describe the kinds of treatment you have received, what did you do in treatment, do you know what the treatment was called?**

**Do you feel like your responses have been influenced by the treatment you have received? Or by media you've consumed (e.g. books, forums)?**

Thank you for sharing these reasons. This type of research really helps us to better understand how we can help people in the future. We also have an ongoing list of functions on the survey so that you can check off all of the reasons that apply to you. This will help us get a sense of any functions you might have forgotten and what functions are not relevant to you.

**Would you please complete this checklist now and let me know when you have finished?**

Participant completes checklist:

Thank you for completing that. This is the end of the study. Would you like to be entered into our draw to win one of six prizes for participating? If so, I will enter your information into a password-protected document, separate from your data. Once we have fully completed our data collection we will draw names from this list and contact you if you are selected.

Clinician Interview Script:

We are interested in learning more about the reasons why people engage in eating disorder behaviours, such as restricting, binge eating, and purging. In order to assess these reasons, or the functions that eating disorders may serve, we are developing a measure that will ask individuals to check off and rate all functions that are relevant to them. Before we make the list of these functions, we want to ask you about your perspective on why individuals engage in eating disorder behaviours.

**What do you think are the reasons people with eating disorders engage in the following behaviours: restriction, binge eating, self-induced vomiting, laxative use, excessive exercise?**Clinician responds:

**Prompt (if necessary):** Examples of these reasons might be to lose weight, to regulate emotions, or to punish oneself.

**Do you think the reasons your patients/clients believe they engage in these behaviours is different from what you have listed? If so, please describe what those differences are.**

**Finally, do you think that the functions of eating disorder behaviours has changed for patients during COVID-19? That is, do you think individuals might engage in these behaviours for different reasons during this time?**

Clinician responds Yes or No:

If Yes: **How do you think these functions have changed?**

Clinician responds:

**What treatments do you most often use for individuals with eating disorders?**

Thank you. Now we have an ongoing list of functions on the survey so that you can check off all of the functions that you think are relevant to eating disorder behaviours. This will help us get a sense of any functions you might have forgotten or what functions you think are not relevant at all.

**Would you please complete this checklist now and let me know when you have finished?**

Thank you for completing that. This is the end of the study. Would you like to be entered into our draw to win one of six prizes for participating? If so, I will enter your information into a password-protected document, separate from your data. Once we have fully completed our data collection we will draw names from this list and contact you if you are selected.
